# Supplementary material for: Electrocorticography is superior to subthalamic local field potentials for movement decoding in Parkinson’s disease
Source: eLife. 2022 May 27;11:e75126. doi: 10.7554/eLife.75126 (PMC9142148; doi:10.7554/eLife.75126)
Supplement: Supplementary file 1. — (a) Electrode Details (b) Bayesian Optimization Hyperparameters (c) Best channel R2 performances. [file elife-75126-supp1.docx]

**Supplementary File 1A: Electrode Details.**

| **Name** | **Location** | **Number of contacts** | **Layout** | **Contact Area [mm]** | **Shape** | **Contact Length [mm]** | **Contact diameter [mm]** | **Maximum observed decoding performance (single channel)** | **Maximum observed decoding performance (combined channels)** |
| --- | --- | --- | --- | --- | --- | --- | --- | --- | --- |
| Medtronic DBS Electrodes 3389 | STN | 4 | 1x4 | 5.89 | cylindric | 1.5 | 1.27 | 0.68 | 0.66 |
| AD-Tech IS06R-SP10X-000 | ECoG | 6 | 1x6 | 12.56 | circular | - | 4 | 0.71 | 0.8 |
| AD-Tech IS08R-SP10X-000 | ECoG | 8 | 1x8 | 12.56 | circular | - | 4 | 0.37 | 0.29 |
| AD-Tech FG28A-MP04-0T4 | ECoG | 28 | 2x16 | 3.14 | circular | - | 2 | 0.64 | 0.54 |

**Supplementary File 1b: Bayesian Optimization Hyperparameters**

| **Hyperparameters** | **Elastic Net Regularized Wiener Filter** |
| --- | --- |
| Time lag | 500 ms |
| L1 ratio alpha | 0 to 1 |
| L1/L2 ratio rho | 0 to 1 |
| iterations | 1000 |
| used library | sci-kit learn |
|  |  |
| **Hyperparameters** | **Neural Networks** |
| input nodes | 1 to 10 uniform |
| dense layers | 1 to 3 uniform |
| dense neurons | 1 to 10 uniform |
| activation function | sigmoidal and tanh |
| dropout factor | 0.2 |
| Adam learning rate | 0.0001 to 0.01 log uniform |
| Adam beta 1 | 0.9 |
| Adam beta 2 | 0.999 |
| eta | 0.999 |
| batch size | 100 |
| epochs | 1000 |
| loss function | MSE |
| val split | 0.8 |
| early stopping epochs | 10 |
| used library | tensorflow |
|  |  |
| **Hyperparameters** | **XGBOOST** |
| boosting rounds | 10 |
| depth | 1 to 10 uniform |
| eta tree scaling | 0.00001 to 1 uniform |
| splitting loss regularization | 1 to 10 uniform |
| max depth | 6 |
| min child weight | 1 |
| max delta step | 0 |
| sampling method | uniform |
| L1 regularization | 0 |
| L2 regularization lambda | 1 |
| num parallel trees | 1 |

**Supplementary File 1c: Best channel** $\boldsymbol{R}^{\boldsymbol{2}}$ **performance**

| **ML method** | **ECoG**  **Con.** | **ECoG**  **Ips.** | **STN**  **Con.** | **STN**  **Ips.** |
| --- | --- | --- | --- | --- |
| Elastic - Net | 0.14±0.1 | 0.06±0.04 | 0.06±0.11 | 0.03±0.03 |
| Wiener Filter | 0.19±0.13 | 0.09±0.06 | 0.1±0.16 | 0.067±0.087 |
| Neural Networks | 0.14±0.24 | 0.09±0.19 | 0.015±0.04 | 0.0±0.0 |
| SPoC + Elastic - Net | 0.16±0.12 | 0.085±0.07 | 0.09±0.16 | 0.05±0.068 |
| SPoC + XGBOOST | 0.23±0.2 | 0.09±0.14 | 0.09±0.2 | 0.05±0.11 |
| XGBOOST | 0.31±24 | 0.13±0.16 | 0.09±0.2 | 0.07±0.1 |
